# Patient-centred interprofessional teleconsultation for post-viral symptom complexes in German primary care—protocol for the cluster-randomised controlled COVI-Care M-V trial

**DOI:** 10.1186/s12913-025-13276-6

**Published:** 2025-09-09

**Authors:** Christin Löffler, Anne Daubmann, Daniela Endlicher, Viacheslav Galchenko, Ralf Jendyk, Emil Christian Reisinger, Martina Sombetzki, Ann-Kathrin Ozga, Anja Wollny, Gregor Feldmeier

**Affiliations:** 1 Institute of General Practice, Rostock University Medical Center, Doberaner Str. 142, Rostock, 18057 Germany; 2 Institute for Medical Biometry and Epidemiology, University Medical Center Hamburg-Eppendorf, Martinistraße 52, Hamburg, 20246 Germany; 3 MEYTEC GmbH Medizinsysteme, Akazienstr. 13, Werneuchen OT Seefeld, 16356 Germany; 4 Department of Tropical Medicine, Infectious Diseases and Nephrology, Rostock University Medical Center, Schillingallee 35, 18057 Rostock, Germany

**Keywords:** Post-Acute COVID-19 Syndrome, Fatigue Syndrome, Chronic, Telemedicine, Primary Health Care, Patient-Centered Care, Rural Health Services

## Abstract

**Background:**

Post-viral syndromes, including long- and post-COVID, often lead to persistent symptoms such as fatigue and dyspnoea, affecting patients' daily lives and ability to work. The COVI-Care M-V trial examines whether interprofessional, patient-centred teleconsultations, initiated by general practitioners in cooperation with specialists, can help reduce symptom burden and improve care for patients.

**Methods:**

To evaluate the effectiveness of the intervention under routine care conditions, a cluster-randomised controlled trial is being conducted. The primary outcome is physical performance, assessed by the difference between the actual and individually defined target distance in the 6-min walk test. Secondary outcomes include health-related quality of life (PAC-19QoL), cognitive function (DemTect), lung function (peak flow meter), fatigue symptoms (FAS), and weekly working hours, measured at baseline (T0) and after four months (T1).

**Results:**

In the absence of evidence-based therapies for post-viral symptom complexes, multidisciplinary care and support currently represent the mainstay of patient management. The COVI-Care M-V study evaluates a regionally tailored, patient-centred intervention, developed based on qualitative research and piloting. Anticipated challenges include selection bias, recruitment difficulties due to healthcare staff shortages, and underreporting of symptoms, which will be addressed through targeted awareness materials and digital support for participating practices.

**Conclusions:**

This study aims to generate evidence to enhance care, particularly by optimising healthcare structures for affected individuals.

**Trial registration:**

ISRCTN, ISRCTN11050086. Registered 28 April 2025

**Supplementary Information:**

The online version contains supplementary material available at 10.1186/s12913-025-13276-6.

## Contributions to the literature

• Illnesses after a viral infection, like post-COVID, can cause many symptoms, such as tiredness or trouble breathing. Because these symptoms vary so much, it is often difficult for doctors to diagnose the condition.
• Apart from individual therapeutic approaches that alleviate individual symptoms, there are currently no medications available to treat the disease.
• Currently, the best way to support patients is through care from different types of healthcare professionals working together.
• This study looks at whether video consultations, where general practitioners and specialists team up to help patients, can reduce symptoms and improve quality of life.

## Background

Post-viral syndrome including long- and post-COVID refers to a range of health conditions that patients suffer from after surviving various viral infections, including flu, a SARS-Cov-2 primary infection, and others [1]. Although the exact prevalence of long- and post-COVID cannot yet be clearly determined for Germany due to a lack of population-representative studies, studies point in the same direction. According to the German study by Peter et al. (2022), the estimated prevalence of post-COVID is at least 6.5%, 6 to 12 months after a SARS-Cov-2 infection [2]. Unfortunately, more recent representative data do not exist. The most common long-term symptoms include persistent fatigue and dyspnoea, as well as cardiovascular and neurological complaints. Four symptom complexes from the pulmonary, cardiological, angiological, and psychological areas are often distinguished [3]. Symptoms may persist, recur, or fluctuate after initial recovery from acute SARS-CoV-2 infection [4]. Long- and post-COVID is thus a complex condition with long-lasting, heterogeneous symptoms [5]. Especially for people in employment, the condition can pose a major challenge, making a (full) return to work considerably more difficult. The same applies to patients with other post-viral conditions, especially myalgic encephalomyelitis/chronic fatigue syndrome (ME/CFS). ME/CFS patients not only experience significant physical weakness (fatigue) that severely limits their activities, but also neurocognitive and immunological symptoms. Before the COVID-19 pandemic, the number of patients in Germany was estimated at around 250,000 [6]. ME/CFS often occurs after an infectious disease, with various pathogens such as Epstein-Barr virus and influenza known to trigger it. During the COVID-19 pandemic, it became apparent that a subset of patients with long- and post-COVID also develop ME/CFS [7].

In Germany, the interdisciplinary S1 guideline Post-COVID/Long-COVID from the Association of the Scientific Medical Societies in Germany (Arbeitsgemeinschaft der Wissenschaftlichen Medizinischen Fachgesellschaften, AWMF) offers initial guidance on diagnostic and therapeutic options [8]. Furthermore, comprehensive treatment of those affected requires interprofessional structures and networks, which so far only exist to a limited extent in Germany. This applies particularly to rural and structurally weak regions. Patients experience bottlenecks in diagnosis, further treatment and outpatient follow-up care after rehabilitation stays. The Long-COVID Initiative of the German Federal Ministry of Health is currently funding a series of research projects aimed at optimising the care of those affected. The COVI-Care M-V study presented here is also being financed by these funds.

## Methods

### Aim

The COVI-Care M-V cluster-randomised controlled trial investigates the extent to which interprofessional, patient-centred teleconsultation, initiated and supervised by the general practitioner (GP), helps to reduce symptoms of patients with long- and post-COVID and other post-viral symptom complexes. The intervention focuses on the interprofessional exchange between the GP and the patient with specialists for long- and post-COVID at the Rostock University Medical Center.

### Trial design

To examine the effectiveness of the intervention in routine care, we are conducting a cluster-randomised controlled intervention study. Primary outcome is physical performance, measured as the difference between the actual value and an individually defined target value for the walking distance in the 6-min walk test [9]. Secondary endpoints include health-related quality of life (PAC-19QoL) [10], cognitive impairment (DemTect) [11], lung function (peak flow meter) [12], fatigue symptoms (FAS) [13], and weekly working hours. These will be measured before the intervention (T0) and four months later (T1). The trial description is structured in accordance with the CONSORT Statement [14].

### Setting

Mecklenburg-Western Pomerania is a predominantly rural and partly economically weak region with small and medium-sized towns. There are hardly any metropolitan urban centres. In a Germany-wide comparison, demographic aging is particularly advanced here and has been leading to an increase in older patients with chronic diseases and multimorbidity for years. This is accompanied by a decline in the number of healthcare professionals, which is currently reflected in a reduction in health care services. Specialist medical care close to home is not equally accessible to all patients [15]. In particular, specialised outpatient clinics for post-viral symptom complexes are very limited. However, even in large cities, it is not always possible for those affected to see specialists due to their fatigue symptoms.

### Recruitment

The recruitment of physicians will be carried out by writing to GPs in private practice in Mecklenburg-Western Pomerania. For this purpose, a complete list of the Association of Statutory Health Insurance Physicians in Mecklenburg-Western Pomerania will be used. Enough GPs will be contacted in waves until the required number is reached. In addition, existing networks such as the teaching and research networks of the Institute of General Practice at Rostock University Medical Center will be used. Patients will be selected consecutively in the participating practices based on the inclusion and exclusion criteria below. Posters will also be used in GP practices to support patient recruitment and to motivate patients to proactively approach their GP about the study. Studies suggest that some of those affected do not address existing symptoms with their GP for a variety of reasons [16]. After being informed by the participating GPs and signing the declaration of consent, patients are included in the study.

### Inclusion and exclusion criteria

The study will include at least 42 GPs and their patients suffering from post-viral symptom complexes. Patient-related inclusion criteria are the presence of a post-viral symptom complex and an age of at least 18 years.

We will include patients with sequelae of viral infections, with consideration given to the following ICD codes:

- U09.9! Post-COVID-19 condition, unspecified
- U10.- Multisystem inflammatory syndrome associated with COVID-19
- J10.- Influenza due to identified influenza virus
- J11.- Influenza, virus not identified
- U07.1! COVID-19, virus detected
- U07.2! COVID-19, virus not detected
- B27.- Infectious mononucleosis
- G93.3 Chronic fatigue syndrome [CFS], including post-viral (chronic) fatigue syndrome
- J06.9 Acute upper respiratory infection, unspecified

Furthermore, to be included, patients must exhibit at least three symptoms from different symptom complexes:

1. Neurological/Neuromuscular/Psychiatric:
  G93.3 Chronic fatigue syndrome
  F06.7 Cognitive impairment
  U51.- Cognitive dysfunction
  F51.9 Nonorganic sleep disorder, unspecified
  M79.1 Myalgia
  M25.5 Joint pain
  M62.81 Muscle weakness (generalised)
  R51 Headache
  U50.- Motor dysfunction
  F43.- Reaction to severe stress and adjustment disorders
  R26.- Gait and mobility disorders
  F41.9 Anxiety disorder, unspecified
  F32.- Depressive episode
  F43.- Reaction to severe stress and adjustment disorders
2. Cardiovascular:
  I47.9 Paroxysmal tachycardia, unspecified
  I95.1 Orthostatic hypotension
  R00.2 Palpitations
  R42 Dizziness
3. Pulmonary:
  R06.0 Dyspnoea
  U69.6 Chronic cough
  R07.1 Chest pain during breathing
4. Other:
  R43.- Disorders of smell and taste
  R10.4 Other and unspecified abdominal pain
  L65.- Hair loss
  K59.- Other functional bowel disorders
  T78.1 Other food intolerance, not classified elsewhere

Patient-related exclusion criteria are the presence of an acute malignant underlying disease, a remaining life expectancy of less than 12 months as estimated by the GP, language barriers (e.g. hearing impairment, insufficient language skills) or dementia and the lack of capacity to consent.

### Intervention

The intervention focuses on the interprofessional exchange between the GP and patient with a specialist from the Long-COVID-Board at the Rostock University Medical Center using teleconsultation. The interdisciplinary Long-COVID-Board regularly discusses individual patient cases, quality of outcomes, and established diagnostic and treatment procedures, to develop and share best practice models. On the one hand, this approach enables patients to receive care close to home, in familiar structures. On the other hand, a specialist re-evaluates the diagnosis and therapy of the included patients based on specific experience and expertise in the treatment of post-viral symptom complexes. Various symptoms in the pulmonary, cardiological, angiological and neurological areas can thus be considered holistically and further procedures in diagnostics and therapy can be discussed. Each appointment is planned to last 45–60 min and is prepared by the GP in advance by sending the patient report. At the same time, patients are actively involved in the decision-making process. During the teleconsultation, they are on-site in the GP's practice together with their GP on the screen and personally report their complaints and symptoms. They are actively involved in the subsequent consideration of further diagnostic and therapeutic steps. Before the intervention begins, all GPs in the intervention group receive two online training sessions. A 120-min training session addresses current therapeutic approaches in the treatment of post-viral symptom complexes and is conducted by specialists from the Long-COVID-Board. A second training session, which lasts about 90 min, is dedicated to the communicative aspects of conducting teleconsultations and the associated effects on the doctor-patient relationship. Also, techniques of patient-centred communication are taught here. The participating doctors receive an expense allowance per patient. See Tables 1–3 in the supplement for detailed information.

### Technical solutions

This study integrates standardised data collection and technically robust as well as secure teleconsultation service for patients with post-viral symptom complexes in primary care. Both study groups utilise uniform technical setups, centred on the MEYCARE® Set, which includes a tablet personal computer, Bluetooth-enabled medical devices, and an internet connection via a multi-SIM (Subscriber Identity Module) LTE (Long-Term Evolution) network, ensuring optimal connectivity even in rural practices. Data collection is streamlined through the digital trial platform (PDE—Process Development Execution), enabling consistent instrumental measurements and patient self-reports via a digitised questionnaire. All data are securely processed and stored in MEYCLOUD, certified under DIN EN ISO/IEC 27001, using highly regulated access via a VPN (Virtual Private Network) tunnel and encrypted data sharing. MEYTEC provides operational support, including replacement devices to minimise downtime. Teleconsultations use the MEYDOC® solution for end-to-end encrypted audiovisual communication, with tailored training provided to participating practices.

### Randomisation and control group

All participating practices will be randomly assigned to the intervention or control group in a 1:1 ratio centrally by the Institute for Medical Biometry and Epidemiology at the University Medical Center Hamburg-Eppendorf. Block-randomisation with variable block length will be used. Patients’ baseline (T0) characteristics can only be collected after randomisation of GPs but before start of intervention.

The participating patients of a practice are thus either completely in one group or the other (cluster-randomisation). Contamination effects are thus avoided. Patients in the control group receive conventional care (care as usual) and an information brochure about post-viral symptom complexes. After data collection, general practitioners in the control group will have the opportunity to participate in both online training courses on post-viral symptom complexes and communicative aspects of teleconsultations. This is to prevent practices from dropping out of the study prematurely. Physicians in the control group will also receive compensation for each included patient.

### Outcomes

The primary endpoint is physical performance capacity, measured as the difference between the actual value and an individually defined target value of the walking distance achieved in the 6-min walk test. Patients are asked to walk as far as possible in six minutes. In this time, healthy people can cover a distance of 700–800 m [9]. The six-minute walk test is supplemented by measurements of pulse, blood pressure and oxygen saturation. The distance covered is GPS-tracked (Global Positioning System) using an app from MEYTEC GmbH, making it easy to record and implement in the GP practice.

The secondary endpoints include:

1. The PAC-19QoL to assess health-related quality of life. The PAC-19QoL was developed with long- and post-COVID patients in the UK and captures various dimensions [10]. Overall, it consists of 44 items. The developers of the questionnaire provided us with the British original for a professional translation.
2. Lung function, specifically the maximum possible flow rate of air when breathing out, measured using a peak flow meter [12].
3. Symptoms of fatigue are measured using the Fatigue Assessment Scale (FAS) [13].
4. Cognitive impairment is recorded using DemTects [11]. The instrument is already well-known to many GP practices from previous studies.

The following endpoints are digitally recorded and stored by the medical assistant in the practice using hardware and software from MEYTEC GmbH: six-minute walk test, pulse, blood pressure and oxygen saturation measurements, peak flow meter measurements, FAS and DemTect. In addition, patient-related socio-demographic characteristics such as age, gender, marital status, school and vocational training, employment, weekly working hours and comorbidities are recorded. The latter data as well as the PAC-19QoL will be collected directly afterwards (if necessary, also at a second appointment) from the patients via tablet. All primary and secondary outcomes will be recorded at both T0 and T1.

### Sample Size

**Fig. 1:**
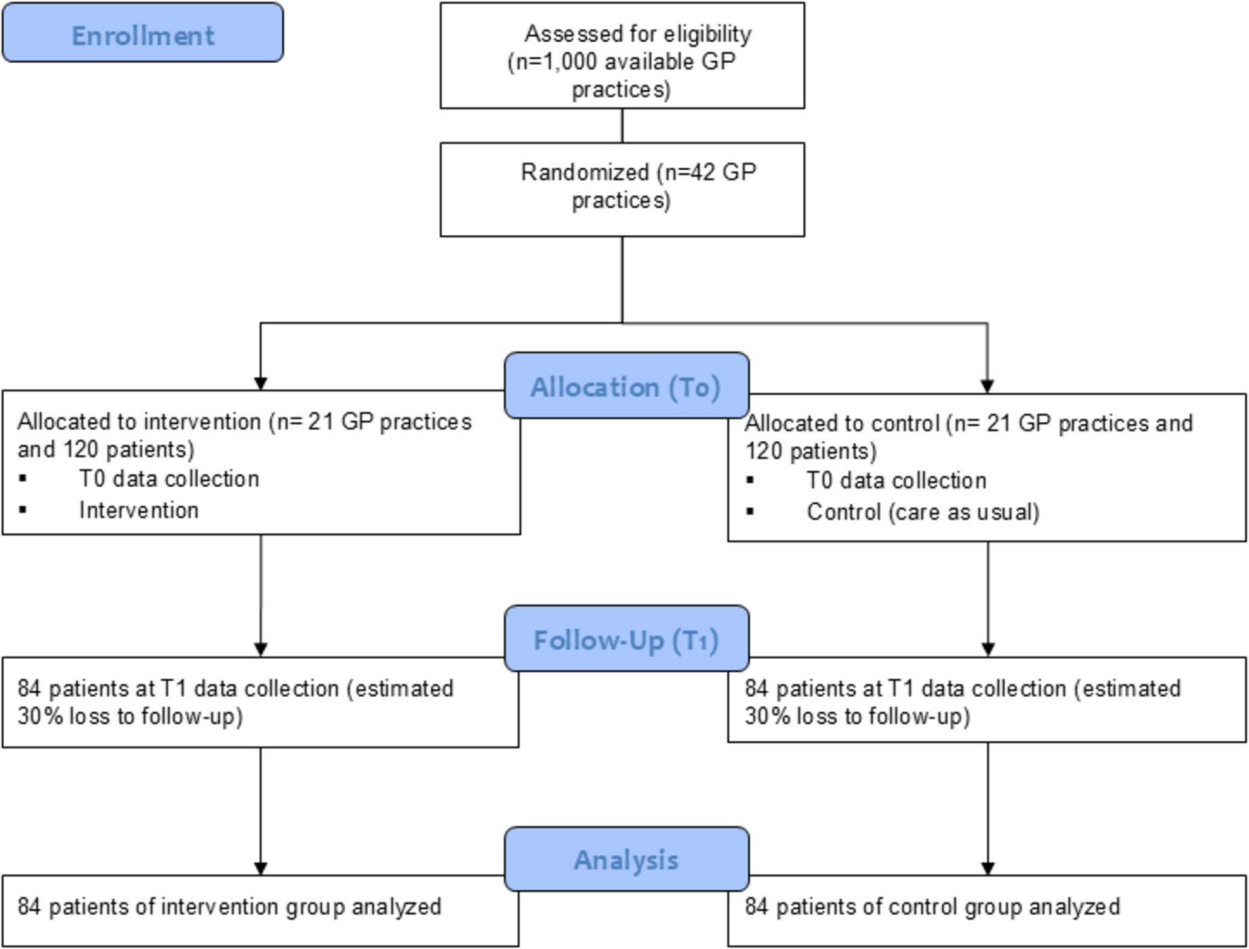
CONSORT flow diagram of the COVI-care M-V trial

We assume that a change in the difference between the actual and an individually defined target of the walking distance of the 6-min walk test from T0 to T1 of 10% can be achieved between the intervention and control group (83% in the control group and 93% in the intervention group). We further assume a standard deviation of 19% in both groups [17]. Furthermore, we assume that 4 evaluable patients will be available per GP practice and that the intra-cluster correlation coefficient is 15%. To be able to demonstrate this difference with a power of 80% and a type 1 error of 5% for a two-sided hypothesis, a total of 42 GP practices (21 practices per group) must participate in the study. This means that 84 evaluable patients (168 patients in total) participate in the study per group. To compensate for a 30% drop-out rate at the patient level, a total of 240 patients will be recruited in 42 practices. The sample size calculation was performed using PASS 2022, module ‘Tests for Two Means in a Cluster-Randomized Design’. See Fig. 1 for the CONSORT flow diagram.

### Blinding

This study does not involve the blinding of patients and study staff. Data analysts will be blinded partially.

### Statistical methods

The patients’ baseline characteristics and GPs characteristics will be described for the total study population and separately for the two groups using mean and standard deviation or median, minimum and maximum and 1 st and 3rd quantile for continuous variables and absolute and relative frequencies for categorical data.

A linear mixed model will be used to evaluate the primary endpoint, defined as the change from baseline of the difference between the actual value and an individually defined target value of the walking distance at T1. Fixed effects will include the baseline value of the difference between the actual value and an individually defined target value of the walking distance and group affiliation, while practice serves as a random effect. Missing values will be imputed using multiple imputation and hence allowing an analysis based on the intention-to-treat population. The mean difference of the group comparison for the primary endpoint at T1 will be reported with its 95% confidence interval and associated p-value (two-sided with significance level = 0.05).

Secondary endpoints will be analysed exploratively without adjustment for multiple testing using linear mixed or generalised linear mixed models (according to the scale level of the endpoint). Planned sensitivity analyses include a complete case analysis (i.e. without imputed data) and a per protocol analysis. Additionally, a multiple linear mixed model for the analysis of the primary endpoint will be conducted including additional covariates of clinical importance (e.g. showing clinically relevant differences between groups) measured at baseline.

Subgroup analyses are planned for the following subgroups: sex, age, education, severity of symptoms at the start of the illness, pre-existing conditions (e.g. depression), rehabilitation (yes/no), and level of urbanisation (rural/urban).

A statistical analysis plan will be finalised before unblinding of statisticians. No interim analysis is planned. Results will be reported and published according to the CONSORT (Consolidated Standards of Reporting Trials) statement for cluster randomised trials [18, 19]. Analyses are carried out with standard software: SPSS, Version 29 or newer (Armonk, NY: IBM Corp.) [20], SAS, Version 9.4 or newer (Cary, NC: SAS Institute Inc.) [21], or R (Version 4.3.3 or newer) [22].

## Discussion

The COVI-Care M-V cluster-randomised controlled trial examines the effectiveness of interprofessional, patient-centred teleconsultations, initiated and guided by GPs, in alleviating symptoms in patients with long- and post-COVID as well as other post-viral symptom complexes. The core of the intervention is the structured interprofessional collaboration between GPs and specialists in long- and post-COVID care at the Rostock University Medical Center, facilitating comprehensive, multidisciplinary patient management.

A recent UK-based Delphi consensus study, conducted with 480 patients and healthcare providers, highlighted key priorities for managing individuals with complex multisystem conditions, including post-COVID syndrome. The study underscored the importance of strengthening integrated care, ensuring access to effective treatments, implementing diagnostic procedures that support personalised treatment within an integrated care framework, and fostering structured collaboration between primary and specialist care [23]. In the absence of an effective therapy for post-viral symptom complexes, multidisciplinary care and support remain the primary approach to managing affected individuals.

However, so far there remains limited evidence on the most effective care structures and approaches for supporting affected patients. The same applies to effective therapies. A 2024 review of 24 trials (n = 3,695) evaluated interventions for long-COVID. Moderate-certainty evidence indicates that cognitive behavioural therapy likely alleviates fatigue and enhances concentration. Supervised physical and mental health rehabilitation may improve overall health, reduce depression, and enhance quality of life. Intermittent aerobic exercise likely benefits physical function [24]. Additional systematic reviews and meta-analyses are currently being conducted to evaluate the efficacy and safety of pharmacological and non-pharmacological interventions for the treatment and management of long-COVID [25].

The COVI-Care M-V study benefits from an intervention that was specifically developed based on findings from a qualitative interview study, ensuring its alignment with regional healthcare structures. Additionally, the study design was pretested in a small pilot study to assess and optimise the feasibility of recruitment and data collection in the primary care setting. The randomised controlled design, which accounts for a 30% dropout rate in its calculations, aims to generate reliable results. Study endpoints are comprehensively defined, incorporating both physical and mental health aspects. A strong emphasis on the patient-centred outcome of health-related quality of life further enhances the study’s design and its overall validity. To achieve the required sample size, an extended recruitment period of two years is planned. Moreover, participating practices benefit from optimised digital data collection and continuous support from the study team.

Despite its strengths, the study is subject to certain limitations. Participation will likely be skewed toward practices with a higher affinity for technology, while those less inclined toward healthcare innovation may be underrepresented. Due to the complexity of the intervention and its requirements, neither the GPs nor the patients can be blinded. The allocation of patients cannot be concealed. Additionally, the healthcare landscape in Mecklenburg-Western Pomerania presents challenges for study participation, including physician shortages, limited practice staff, and an increasing patient burden due to demographic shifts. Preliminary studies indicate that some affected individuals do not always disclose post-COVID symptoms to their GP for various reasons. To address this, educational brochures and practice posters will be utilised to raise awareness. Furthermore, some patients included in the study may already be receiving specialist care.

This study aims to generate evidence to enhance care, particularly by optimising healthcare structures for affected individuals.

## Conclusions

The COVI-Care M-V study investigates the effectiveness of interprofessional, patient-centred teleconsultations in improving symptoms and quality of life for individuals with long- and post-COVID as well as other post-viral syndromes. Given the heterogeneous and often debilitating nature of these conditions, a multidisciplinary approach remains the primary means of providing care in the absence of a definitive treatment. The study design incorporates insights from qualitative research and a pilot study, ensuring alignment with regional healthcare structures and feasibility in routine practice.

## Supplementary Information

Supplementary Material 1: Table 1. Interprofessional teleconsultations (based in TIDieR Checklist) [26]. Table 2. Online training session – post-viral symptom complexes. Table 3. Online training session 2 – communicative aspects of teleconsultations. 

## Data Availability

No datasets were generated or analysed during the current study.

## Abbreviations

AWMF: Arbeitsgemeinschaft der Wissenschaftlichen Medizinischen Fachgesellschaften
CONSORT: Consolidated Standards of Reporting Trials
DemTect: Dementia Detection
FAS: Fatigue Assessment Scale
GP: General Practitioner
GPS: Global Positioning System
ICD: International Statistical Classification of Diseases and Related Health Problems
LTE: Long Term Evolution (mobile communications standard)
ME/CFS: Myalgic Encephalomyelitis/ Chronic Fatigue Syndrome
PAC-19QoL: Post-Acute (Long) COVID-19 Quality of Life Instrument
PDE: Process Development Execution
SARS-Cov-2: Severe Acute Respiratory Syndrome Coronavirus 2
SIM: Subscriber Identity Module
TIDieR: Template for Intervention Description and Replication
VPN: Virtual Private Network
