# Supplementary material for: Patient-centred interprofessional teleconsultation for post-viral symptom complexes in German primary care—protocol for the cluster-randomised controlled COVI-Care M-V trial
Source: BMC Health Serv Res. 2025 Sep 9;25:1194. doi: 10.1186/s12913-025-13276-6 (PMC12418605; doi:10.1186/s12913-025-13276-6)
Supplement: Supplementary file 1 — Supplementary Material 1: Table 1. Interprofessional teleconsultations (based in TIDieR Checklist) [26]. Table 2. Online training session – post-viral symptom complexes. Table 3. Online training session 2 – communicative aspects of teleconsultations. [file 12913_2025_13276_MOESM1_ESM.docx]

**Table 1 Interprofessional teleconsultations (based in TIDieR Checklist)** [26]

| *Item number* | *Item* | *Information* |
| --- | --- | --- |
| *1* | *Name* | Interprofessional teleconsultation involving the patient, GP, and a specialist for post-viral symptom complexes. |
| *2* | *Rationale* | The interprofessional teleconsultation in the presence of the patient should (1) shorten the time to specialised treatment, (2) save patients long journeys, (3) enable consultation in the familiar GP setting, (4) involve the GP in all diagnostic and therapeutic decisions and (5) thereby indirectly develop GP skills in the treatment of patients with post-viral symptom complexes. |
| *3* | *Material* | A standalone hardware and software solution, independent of the practice software, is provided to practices for conducting teleconsultations. |
| *4* | *Procedures* | Appointments are scheduled through an online platform by the GP or practice staff. The GP prepares the required documents and shares them with the specialist before the consultation. |
| *5* | *Provider* | Rostock University Medical Center |
| *6* | *Mode of delivery* | online |
| *7* | *Location* | GP-practices |
| *8* | *Time* | 45 - 60 minutes |
| *9* | *Tailoring* | Teleconsultations are conducted based on the patient's medical history and presenting symptoms. |
| *10* | *Modifications* | Modifications are not planned. |
| *11* | *Intervention adherence* | Adherence to the intervention is assessed through qualitative and quantitative evaluations. |

**Table 2 Online training session – post-viral symptom complexes**

| *Item number* | *Item* | *Information* |
| --- | --- | --- |
| *1* | *Name* | Online training session for primary care physicians to present current approaches to diagnosing and treating post-viral symptom complexes. |
| *2* | *Rationale* | The dissemination of up-to-date information on best practices, including diagnostic methods and treatment strategies. |
| *3* | *Material* | Presentation slides |
| *4* | *Procedures* | The format is primarily lecture-based, with ample time allocated at the end for discussion and questions. |
| *5* | *Provider* | Rostock University Medical Center |
| *6* | *Mode of delivery* | online |
| *7* | *Location* | - |
| *8* | *Time* | 120 minutes |
| *9* | *Tailoring* | To ensure the online training meets the target group's specific requirements, time will be reserved at the end of the training for questions and interactive discussion. |
| *10* | *Modifications* | Modifications are not planned. |
| *11* | *Intervention adherence* | All intervention practices are kindly requested to attend the event. The participant registry will offer insights into the engagement rate of intervention practices. |

**Table 3 Online training session 2 – communicative aspects of teleconsultations**

| *Item number* | *Item* | *Information* |
| --- | --- | --- |
| *1* | *Name* | Online training session for primary care physicians to teach essential communication tools for teleconsultations. |
| *2* | *Rationale* | Online training for general practitioners to familiarise them with the communicative aspects of conducting teleconsultations and the associated effects on the doctor-patient relationship. In addition, techniques of patient-centred communication are taught here. |
| *3* | *Material* | Presentation slides |
| *4* | *Procedures* | This interactive format fosters a collaborative learning environment through the exchange of experiences, facilitated discussions, and hands-on practice of communication techniques. |
| *5* | *Provider* | Rostock University Medical Center |
| *6* | *Mode of delivery* | online |
| *7* | *Location* | - |
| *8* | *Time* | 90 minutes |
| *9* | *Tailoring* | The interactive design will allow for the content to be adapted and delivered in a manner that directly addresses the target group's specific context. |
| *10* | *Modifications* | Modifications are not planned. |
| *11* | *Intervention adherence* | Adherence to the intervention is assessed through qualitative and quantitative evaluations. |
